# Sensor-Aware Recognition and Tracking for Wide-Area Augmented Reality on Mobile Phones

**DOI:** 10.3390/s151229847

**Published:** 2015-12-10

**Authors:** Jing Chen, Ruochen Cao, Yongtian Wang

**Affiliations:** 1 School of Optoelectronics, Beijing Institute of Technology, Beijing 100081, China; chen74jing29@bit.edu.cn (J.C.); wyt@bit.edu.cn (Y.W.); 2 School of Computer Science and Technology, Beijing Institute of Technology, Beijing 100081, China

**Keywords:** mobile augmented reality, sensor-aware scene recognition, VLAD, sensor fusion

## Abstract

Wide-area registration in outdoor environments on mobile phones is a challenging task in mobile augmented reality fields. We present a sensor-aware large-scale outdoor augmented reality system for recognition and tracking on mobile phones. GPS and gravity information is used to improve the VLAD performance for recognition. A kind of sensor-aware VLAD algorithm, which is self-adaptive to different scale scenes, is utilized to recognize complex scenes. Considering vision-based registration algorithms are too fragile and tend to drift, data coming from inertial sensors and vision are fused together by an extended Kalman filter (EKF) to achieve considerable improvements in tracking stability and robustness. Experimental results show that our method greatly enhances the recognition rate and eliminates the tracking jitters.

## 1. Introduction

With the development of mobile devices, mobile phones equipped with high-resolution cameras and multiple sensors are more suitable for augmented reality (AR) applications. Like the traditional augmented reality on PCs, mobile augmented reality (MAR) superimposes registered 3D graphics over users’ view of the real world, allowing users to share the computer’s perception of the environment. In recent years, the entertainment field has been one of the most successful fields at utilizing mobile augmented reality technologies and so far there are a lot of related works [1,2,3,4] which bring users a new experience. For example, ARPP [1] is an AR game built on the Android platform, which can provide a more interesting and convenient way for people to play ping-pong games on their smartphones. CorfuAR [4], a mobile augmented reality travel guide, can help users to find nearby services, such as restaurants and hotels. In those mobile augmented reality systems, one of the challenging existing technical issues is the registration method. Marker-based registration algorithms and later markerless tracking algorithms are utilized to realize robust and highly accurate tracking. However, many research works mainly focus on single target tracking on a small workspace, for example an object on the table or a statue on the square [5]. With the use of small and lightweight mobile devices, mobile AR allows for more unrestricted user movement. Thus, the requirement for wide area tracking capability is becoming increasingly urgent for mobile AR in outdoor environments.

Due to the memory and computation limitations of mobile devices, how to ensure real-time performance in wide-area environments on mobile phones is a challenge. Visual Simultaneous Localization and Mapping (SLAM) is a promising real-time structure and motion approach, which can build a global 3D map covering the whole observed scene to realize registration in wide-area environments [6,7,8]. In particular, a parallel SLAM-based tracker that can build the model of the environment on the fly on mobile phones has been proposed [8]. It can work in small workspaces. However, due to the complexity of natural scenes and the expansion of the system’s workspace, it is difficult to achieve real-time performance on mobile devices. Meanwhile for wide and unconstrained environments pose estimation may be infeasible due to the difficulties of efficiently matching a given image with the whole database of the complete environment. Subsequent attempts [9,10] advanced the methodology further towards a highly robust detection and tracking framework. Modified SIFT and ferns approaches are designed for fast and efficient feature matching on mobile phones. However, investigations of the contribution of global information, for example the GPS and gravity data obtained directly from mobile phones, to the performance of the vision service have not been considered yet. In addition, it is inconvenient to fulfill time-consuming wide-area localization steps on these low-end mobile devices.

Although vision-based recognition and tracking methods can provide higher accuracy, they usually rely on a model of the environment which is sensitive to illumination, occlusion and viewpoint selection. Fusing vision with non-visual sensor data, on the other hand, can provide us more robust performance under fast motion and tracking failures and provide a spatial context for the improvement of the keyframe recognition component. However, little research on sensor-aware recognition and tracking on mobile phones has been done to date.

In view of the problems above, we have made the following contributions in this paper to the design of a real-time sensor-aware scene recognition and tracking method on mobile phones applicable to large-scale outdoor environments. First, we divide the whole wide-area workspace, such as a whole city, into small sub-areas according to their geographic locations by using a density-based clustering method. The use of sub-areas instead of a global environment lets us reduce considerably the computational complexity of reconstructing the whole scenes, and also made our system more suitable for online implementation, especially with large-scale workspaces. Second, we propose a novel keyframe recognition method which combines gravity orientation clues for visual vocabulary generation to improve the recognition accuracy without a time-consuming geometry verification procedure. Third, data coming from accelerometers, gyroscopes and vision are fused together by an extended Kalman filter (EKF) to achieve dramatic improvements in tracking stability and robustness on mobile phones. The GPS, gravity and inertial sensors embedded on mobile phones not only enable us to provide fast and accurate location results, but also produce a robust estimate of the camera pose before any processing of the image, which makes wide-area localization and tracking possible on mobile phones. Because of the use of all these sensors, we call our method “sensor-aware”.

## 2. Related Work

### 2.1. Mobile Visual Recognition

With the popularization of camera-embedded mobile devices, mobile visual recognition has received a wide range of attention from both academia and industry. The bag-of-features approach presented in [11] and its variants [12,13,14] are some commonly used approaches. Given an image, keypoint features are detected and quantized to a visual word, which will be employed to represent an image. An inverted index file is build up to implement visual words-based indexing and searching. For efficient queries these methods need the original feature vectors stored in memory, which will quickly lead to storage and computational problems in most implementations. Some approaches have been proposed to compress the tree histogram [15] or inverted files [16] to solve the storage problem in the mobile phone’s limited memory. However, both methods require some selective decompression during a query process. Compressed Fisher vector [17], VLAD [18] and REVV [19] are adopted for efficient data organization and search. Database image representations are generated from local descriptors like SIFT [20], PCA-SIFT [21] or SURF [22], yet they utilized visual word residuals aggregation to replace bag-of-words histograms which can utilize a much smaller codebook and perform comparisons directly in the compressed domain. Such a small codebook reduces the memory requirements of the vector quantization and makes it possible to run on a mobile phone platform. VLAD can be seen as a simplified non-probabilistic version of the FV and it is faster to compute. Many mobile visual recognition systems are based on VLAD and its variants [23,24,25]. There are also plenty of applications utilizing those mobile visual recognition methods in the field of entertainment. For example, Layar [26], an augmented reality browser, provides readers a new way to “read” magazines. Once people use their smartphones to scan magazines, Layar will show them more about what they read such as videos, websites or 3D models. SyFy TV, a channel of Junaio [27], lets people see different images pop up on their mobiles’ screens when they point their phones toward advertisements.

While promising, there are still some problems to be solved to further improve the recognition accuracy. For example, geometric information which is proved to be useful for improving retrieval accuracy is neglected absolutely. Besides mobile phones commonly provide additional sensors which can also be used to facilitate the visual recognition process.

### 2.2. Camera Tracking on Mobile Phone

In the past decades, real-time camera tracking technology applied for augmented reality systems has gone from marker-based tracking to the current stage of markerless and hybrid tracking methods. In recent years, natural feature-based real time camera tracking has been extensively studied. Visual structure from motion (SfM) and simultaneous localization and mapping (SLAM) are two kinds of prevalent techniques that have been used for wide-area camera tracking. While SfM has been rooted in the off-line optimal reconstructions of the scene structure and camera trajectory, SLAM approaches involve recovering the environment structure and the camera pose in a recursive way. As demonstrated in [28], the authors presented a camera tracking system called monoSLAM, which could recover the 3D structure of the unprepared scenes while meeting the real-time requirements. Nevertheless, due to the fact that monoSLAM is a system for PC-based AR and it uses a large amount of memory, it is not suitable for mobile augmented reality. In [29], the authors presented a real-time camera tracking and reconstruction system relying on alignment of every pixel rather than feature extraction. Recently the work presented in [6] proposed to use keyframes to build up local panorama maps registered in the 3D map instead of filtering the corresponding keyframe candidates and running both on mobile phones and a PC. In [30], the authors used orientation information from mobile phones’ inertial sensors to resolve inherent ambiguities for 3D pose estimation when tracking on mobile phones. In order to apply to a wide area, [31] used multiple sub-map-based methods instead of single global map method, which is more suitable for mobile phones. Benefiting from those researches on tracking, there are a lot of related applications, especially AR games [32,33], on the market.

On the other hand, a wealth of research work has reported often enough in the past that hybrid tracking methods can achieve considerable improvement in tracking stability and robustness over either sensor alone. For example, earlier in [34], the authors demonstrated a model-based hybrid tracking system for outdoor augmented reality systems. An edge-based tracker was utilized to estimate the accurate camera pose, with gyroscope measurements to deal with fast motions. The work in [35] described a camera tracking system for AR applications, which fused IMU and camera data in a tightly coupled manner by an error-state extended Kalman filter (EKF). As there is more than one possible combination of fusing inertial sensors data and vision data at an extended Kalman filter (EKF), [36] proved that fusing different sensors’ data in the correction stage would be the best approach. Because mobile phones are equipped with high-resolution cameras and multiple sensors, it is a good choice to make hybrid tracking systems on them. The most common method to fuse visual and inertial sensors data on mobile phones is also the Kalman filter and its variants [37,38,39,40]. Among them, the work in [37,38] fused inertial and visual data from mobile phones by an extended Kalman filter (EKF), while [39] proposed to use an unscented Kalman filter (UKF). In [40], the authors took into account the rolling-shutter effect rather than only assuming that all cameras use a global shutter. After our investigation, we noticed that there are a few research projects on data fusion on mobile phones, most of which only focused on gyroscopes when tracking because of the low accuracy of the accelerometers installed on mobile phones. In our study, we have used the wavelet filter to improve the reliability of accelerometer data based on a number of experiments. As a result, our system can keep tracking for a while even when the visual target is lost.

## 3. Sensor-Aware Recognition and Tracking

### 3.1. System Framework

**Figure 1:**
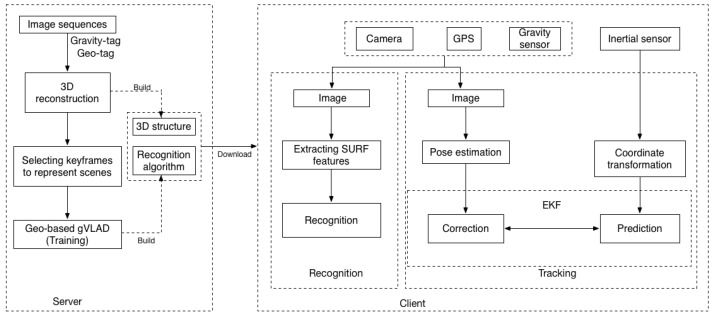
Framework overview.

Our system is based on a client/server architecture. An overview of our framework is given first in Figure 1, which is divided into the offline data processing stage and the online stage. The offline data processing module is responsible for 3D reconstruction of scenes, selecting keyframes and training the keyframe recognition algorithm with geographic and gravity tagged camera captured images. In our algorithm, we partition the whole wide area scene into some geometry independent sub-scenes, and all built sub-scenes are integrated into a tracking system by using our keyframe image recognition algorithm. Images tagged with GPS and gravity information can help us to reduce the image search scope and improve the recognition performance during the online visual recognition process. During the online stage, the geographic location information is used to locate the geographical regions of mobile devices. Gravity information from mobile phones is used to measure the rotation of images with upright direction. A KLT tracker is employed to realize the frame-to-frame tracking of ORB features instead of frame-to-frame matching. Finally, our system combines markerless camera tracking with inertial measurements in an extended Kalman filter framework for optimal pose estimation purposes. It is worth noting that we use SURF features for recognition and ORB features for tracking to ensure the real-time performance on mobile phones.

We will introduce key issues of our system in the following sections. In Section 3.2, we will introduce the offline data processing stage. The 3D structure and recognition algorithm will be described in this section. In Section 3.3, we will introduce our sensor-aware scene recognition algorithm applied to outdoor environments. Gravity information and GPS are used to improve the recognition performance of the VLAD method. On the basis of recognition results, in Section 3.4, data coming from inertial sensors and vision are fused together by an extended Kalman filter (EKF) to achieve dramatic improvements in tracking stability and robustness.

### 3.2. 3D Reconstruction of Scenes

**Figure 2:**
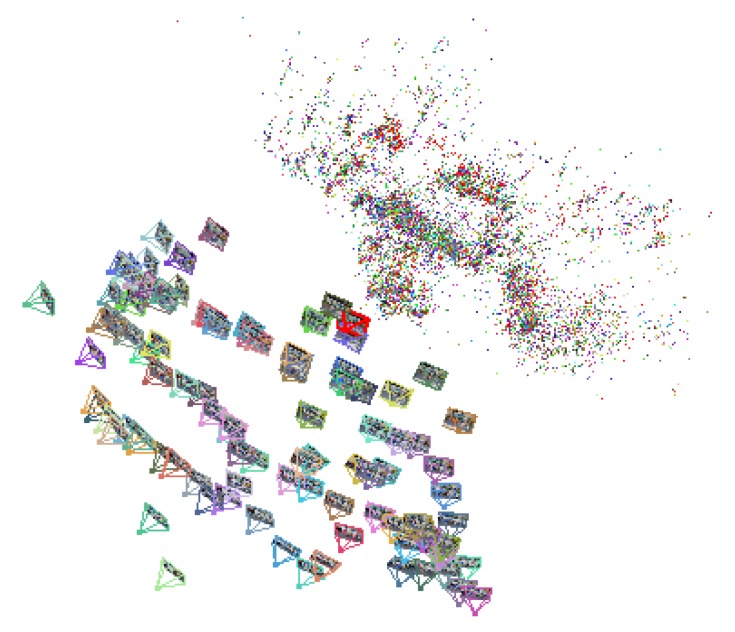
An example of 3D reconstruction of scenes.

During the offline stage, we utilize a camera phone to capture a moderate scale scene from different viewpoints with GPS and gravity tags. Since a GPS device’s sampling rate is about 1 Hz, we use a linear interpolation method to obtain the geometry tag of each input frame. For each scene, a keyframe-based SfM method [41] is employed to build the 3D structure of this scene. Four to five keyframe images containing sufficient and evenly distributed salient feature points are selected as keyframe images. Here we use SQLite to store the 2D/3D correspondence of each point between keyframe images and 3D structures and use a XML file to store feature descriptors of that point. For tracking, once points on the current frame and points stored in the XML file are matched, we can quickly get their corresponding 3D coordinate values from SQLite with the help of 2D/3D correspondence so that the camera pose can be estimated. Once all the needed 3D structures of outdoor scenes are built, we organize all obtained keyframes by using a sensor-aware VLAD algorithm discussed in Section 3.3 for online scene recognition use. With the 3D structure and recognition algorithm built, we can then download them to the mobile phone on which the real time camera tracking will be carried out. One of 3D reconstruction of scenes is illustrated in Figure 2.

### 3.3. Scene Recognition Algorithm for Wide-Area Scenes

This section describes our keyframe image recognition algorithm, which is able to match each online frame to candidate keyframes stored in an image database. Our recognition algorithm uses additional orientation information to make it possible to distinguish features with similar visual appearance at different rotation changes to improve the recognition performance of the VLAD algorithm. With the help of GPS, our system is more suitable for wide-area applications.

#### 3.3.1. VLAD Algorithm

Recently, VLAD has attracted many researchers’ attention because of its speed and scalability. In VLAD, a visual codebook *C* = {*c*_1_ *c*_2_… *c_k_*} of K visual words is learned offline by using a classical K-means approach for all local feature descriptors of training images. The codebook is formally used as a quantization function to assign each d-dimensional local descriptor x to its nearest visual word *c_k_*. Here, we assume that an image is represented by a set of local features as *X* = {*x*_1_, *x*_2_,…, *x_n_*}. A VLAD signature of an image can be obtained by directly concatenating the aggregated residual vector *v_k_* = Σx − *c_k_*, where *c_k_* = *NN*(*x*) and the concatenation vector *v* = [*v*_1_, *v*_2_,…, *v_k_*] is a *K* × *d* dimensional vector. Finally, the concatenation vector *v* = [*v*_1_, *v*_2_,…, *v_k_*] is normalized by power law normalization to avoid the burstness problem.

#### 3.3.2. Gravity-Aware VLAD Algorithm

In the VLAD algorithm, using L2 distance metrics to assign local visual descriptors may cause a situation where those features with similar semantics may be far away from each other, while the features with different semantics may be close to each other. This will unavoidably lead to a decrease in the retrieval performance. To alleviate that problem, we present a gravity-aware VLAD method by taking advantage of dominant orientation information that were already obtained at the feature extraction stage. In this paper, we call it the GVLAD algorithm. For GVLAD, we only cluster features with similar characteristics of orientation to the same visual code.

Finding the absolution rotation direction of images and orientation quantization are the two most important steps of our GVLAD method. The gravity direction θ_*g*_ of a user’s mobile phone can be roughly calculated by using gravity-sensor information *G_i_* = [*g_x_*(*i*), *g_y_*(*i*), *g_z_*(*i*)] as follows:

(1) $$\theta_{g}=\left\{ \begin{array}{lll} \frac{g_{y}\left(i\right)}{\left|g_{y}\left(i\right)\right|}\times \frac{\pi }{2}-\text{atan}\frac{g_{x}\left(i\right)}{g_{y}\left(i\right)} & & \left(g_{y}\left(i\right)\neq 0\right)\\ \frac{\pi }{2}-\frac{g_{x}\left(i\right)}{\left|g_{x}\left(i\right)\right|}\times \frac{\pi }{2} & & \left(g_{y}\left(i\right)=0\right) \end{array}\right.$$

Given all of local visual features *X* extracted from dataset, we firstly construct a gravity-aware codebook by clustering absolute orientation context θ_*angle*_ = |θ_*d*_ − θ_*g*_| of all descriptors with *O_bins_* equally sized orientation regions. Here θ_d_ is the dominant orientation of feature descriptors, θ_*g*_ is the gravity direction of a mobile phone. Typically angles have a circular distribution in the range of [0, 2π), therefore the absolute orientation angle should be calculated as:

(2) $$\theta_{angle}=\left\{ \begin{array}{lll} \left|\theta_{angle}\right| & & else\\ \theta_{angle}+2\pi & & if\;\theta_{angle}<\pi \\ \theta_{angle}-2\pi & & if\;\theta_{angle}<-\pi \end{array}\right.$$

**Figure 3:**
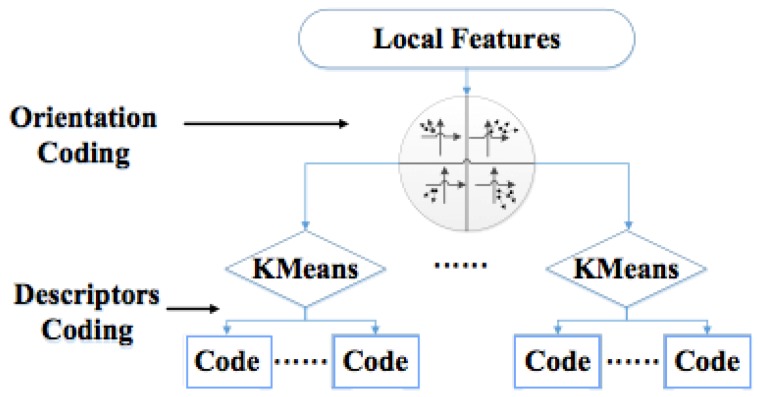
Framework of GVLAD method.

After estimating absolute orientation, we utilize a simple orientation quantization function $\varphi (O(x))=\left\lfloor O_{bins}\times \frac{\theta }{2\pi }\right\rfloor$ to partition local features descriptors *X* into *O_bins_* orientation clusters, here *O* is the index of orientation bins. Figure 3 illustrates the framework of our GVLAD method. Each local feature will be assigned to its visual word according to its geometrical orientation context and descriptor.

Additional orientation information will make it possible to distinguish features of similar visual appearance at different rotation changes. After orientation context-based clustering, we can arrange these visual features pooled in the same bins with any state-of-the-art coding schemes, such as BoW, FV and VLAD.

#### 3.3.3. GPS-Aware GVLAD Algorithm

**Figure 4:**
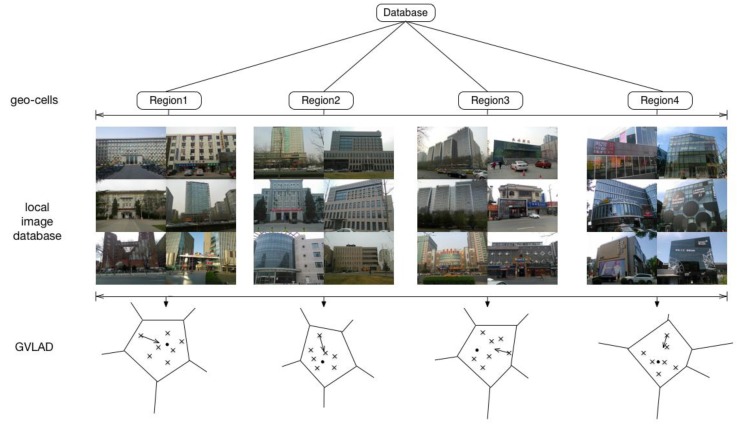
A novel methodology to introduce geo-based GVLAD.

In fact a coarse estimation of the user’s location via GPS can provide sufficient search space information, which can narrow down the image database to a small range. On the basis of GVLAD, we propose a more efficient scene recognition algorithm. We call it the geo-based GVLAD method. At the offline stage, we group database images into geo-cells according to the geometry information measured by a GPS sensor. A density-based clustering method is used to partition the geometry information into different geographical regions. Then, we consider each cluster as a root node of the GVLAD method. Figure 4 gives an illustration of geo-based GVLAD method, where the global map is divided into four geographical regions. One thing that needs to be noted is that the density-based clustering method we used has two parameters to control cluster regions. One parameter is radius and the other is minimal points. By setting different parameters in the density-based clustering method, our method can suit different scale scenes ranging from a small office room to a large city. Meanwhile the geo-based GVLAD allows us to limit irrelevant retrieval data and only consider images coming from nearby locations, which can improve the retrieval speed and recognition rate significantly.

### 3.4. Sensor-Aware Tracking

#### 3.4.1. Pose Estimation

To ensure the real-time operation of the system on mobile phones, here we use ORB features. Given a current image, newly detected ORB features are matched to those features lying on key-frames. A KLT tracker is employed to realize the frame-to-frame tracking. After that, we can establish 2D correspondences between the current image points *m_c_* and the keyframe *m_k_*. Since a feature in the keyframes corresponds to a 3D point in the reconstructed model, we can use these 2D/3D correspondences to estimate camera pose. Given a set of 2D-to-3D matches for current frame t, we can compute the corresponding camera pose parameters by minimizing the re-projection error:

(3) $$\text{min}\sum_{i}{\left\|K\left[R_{t}|T_{t}\right]M_{i}-m_{i}\right\|}^{2}$$

In Equation (3), the camera pose parameters $\left[R_{t}|T_{t}\right]$ are the only unknowns in the vector (assuming the internal camera parameter K fixed). We initialize $\left[R_{t}|T_{t}\right]$ to $\left[R_{t-1}|T_{t-1}\right]$. For the initial frame of the image sequence or the one immediately after tracking failure, keyframe recognition algorithm is used to find the corresponding keyframe.

The solution to Equation (3) can provide a reasonable estimate of the camera pose, yet typically leads to the jitter problem, which is particularly noticeable when the camera is completely or nearly stationary. In order to stabilize the solution, we use the pose estimation results as initial data and add a smoothing term which favors minimum camera motion between consecutive frame sequences to optimize the final pose estimation. Equation (4) shows the cost function:

(4) $$\text{min}\sum_{i}{\left\|K\left[R_{n}|T_{n}\right]M_{i}-m_{i}\right\|}^{2}+\lambda_{1}{\left\|R_{n}-R_{n-1}\right\|}^{2}+\lambda_{2}{\left\|T_{n}-T_{n-1}\right\|}^{2}$$

where λ_1_ and λ_2_ are the different weights on the camera pose parameters. At first, we solve for $\left[R_{t}|T_{t}\right]$ using Equation (1), with λ_1_ = 0, λ_2_ = 0. Once a local minimum has been reached, we execute a few additional Levenberg-Marquardt iterations by solving Equation (4) with gradually updating values of λ_1_ and λ_2_ as follows:

(5) $$\lambda_{1}=\frac{e\left(R_{k}^{n},T^{n}\right)}{\text{min}\left\{e\left(R^{n-1},T^{n-1}\right),e\left(R_{k}^{n},T_{k}^{n-1}\right)\right\}}$$

(6) $$\lambda_{2}=\frac{e\left(R_{k}^{n},T^{n}\right)}{\text{min}\left\{e\left(R^{n-1},T^{n-1}\right),e\left(R_{k}^{n-1},T_{k}^{n}\right)\right\}}$$

where *e*(*R*, *T*) is the re-projection error, n represents the n-th frame image and k is the k-th iteration. In Equation (5), use the translation matrix of the last frame *T*^*n*−1^ and the rotation matrix of the current frame *R^n^* to calculate the re-projection error. If the re-projection error *e*(*R^n^*, *T*^*n*−1^) is small, which shows that the change between two adjacent frames is small, increase the parameter λ_1_. If the re-projection error *e*(*R^n^*, *T*^*n*−1^) is large, which shows that the change between two adjacent frames is large, reduce the parameter λ_1_. The tuning method of parameter λ_2_ is the same as the parameter λ_1_. As a result, larger values of λ_1_ and λ_2_ are used for slower frame-to-frame motions, which can significantly reduce jitter. However when the camera motion is fast or abrupt, the jitter problem is not the major consideration.

#### 3.4.2. Sensor Fusion

**Figure 5:**
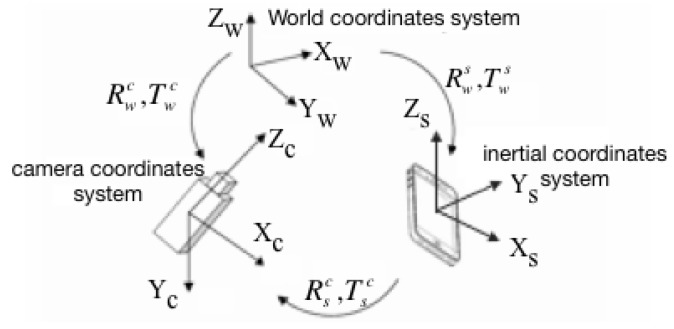
Relative coordinate systems.

In this section, we will fuse a low frequency vision sensor and a high frequency inertial sensor to overcome the limits of any single technology. An extended Kalman filter is used for fusing visual and inertial measurements from camera phone sensors. The geometry and related coordinates to support the development of equations for our sensor fusion problem are illustrated in Figure 5. $\left(R_{s}^{c},T_{s}^{c}\right)$ are the rotation and translation between the camera and inertial sensors, which have been pre-calibrated by using Horn method [42]. Meanwhile the intrinsic parameters of camera are calibrated using the method developed by Zhang [43].

##### Fusion Core

As described in [44], considering the inertial measurements as control inputs to the time update of the Kalman filter can reduce the number of features needed and provide significantly higher prediction quality. Thus, in our fusion model, we only use inertial measurements as control inputs and assume that the camera’s motion has a constant angular velocity and constant acceleration. Therefore the state vector can be represented by $\vec{x}=(q_{s}^{w},\omega ,p_{s}^{w},v_{s}^{w},a,△a)$. Here the unit quaternion $q_{s}^{w}=(q_{0},q_{1},q_{2},q_{3})$ is used to represent the orientation of sensor frame S relative to world frame W. *p* and *v* are the position and velocity of the inertial sensor with respect to the world frame. ω, *a* are the gyroscope and accelerometer, respectively. ∆*a* is accelerometer’s error. For an inertial sensor, accelerometers produce three acceleration measurements *a_s_* (units are m/s^2^). Here *a_s_* is the acceleration in the moving frame, which must be transformed into free acceleration measurements by:

(7) $$a=q_{s}^{w}⊗(a_{s}-g)⊗{\bar{q}}_{s}^{w}$$

In Equation (7), *g* denotes the gravity direction in the world coordinate system and ${\bar{q}}_{s}^{w}$ is the inverse quaternion of $q_{s}^{w}$. Gyroscopes produce three angular velocity measurements ω, one for each axis (units are rad/s), so the system dynamics in the inertial frame at time *t* + Δ*t* can be expressed by the following equation:

(8) $$x_{t+△t}=\left[\begin{array}{c} p_{t}+v_{t}\cdot △t+0.5t^{2}\cdot a\\ v_{t}+△t\cdot a\\ \left[\begin{array}{l} \text{cos}(0.5\omega △t)\\ \sin(0.5\omega △t)\frac{\omega }{\left\|\omega \right\|} \end{array}\right]⊗q_{t}\\ \omega \\ a+△a \end{array}\right]$$

In our sensor fusion model, only vision-based measurements are used to correct the prediction. The orientation and position between camera and world frame at time t can be expressed by:

(9) $$p_{c}^{w}={\left(q_{s}^{w}\right)}^{*}⊗T_{c}^{s}⊗q_{s}^{w}+p,q_{c}^{w}=q_{c}^{s}⊗q_{s}^{w}$$

where $q_{c}^{s}$ and $T_{t}^{s}$ are the rotation and translation between the camera and inertial sensors. Because the orientation and translation data expressed by state vector are represented in the inertial sensor system, the vision measurements data should be transformed into the sensor coordinate system.

##### Failure of Vision Measurements

A failure of the vision measurements occurs easily generated in the case the feature disappears or in case of the mistracked image features. In order to make the motion estimation more robust and applicable, some kind of reliable failure detection is needed. An inertial sensor can assist in stabilizing the camera allowing it to quickly redirect its gaze when motion blurs visual feedback. If no vision measurements are output, the state uncertainty will obviously increase. Therefore we can compute the Frobenius norm of the state uncertainty and compare it to a threshold. Furthermore the translation measurement in the state vector is also checked. If the change of the translation is significant and exceeds the threshold (see Equation (10)), we will use inertial sensor data to update the state vector directly:

(10) $$sqrt(\sum_{i=1,2,3}{\left(p_{t}^{i}-p_{t-1}^{i}\right)}^{2})>threshold$$

where *p_t_* is the translation estimate at time *t* and *p*_*t*−1_ is the translation estimate at time *t* − 1.

## 4. Experimental Results

The system presented in this paper is a C/S architecture. The server is built on a personal computer with an Intel(R) Xeon(R) CPU E5-2670 @ 2.60 GHz and 8 G RAM. The client is built on an iPhone5, which is equipped with a camera, GPS, gravity and inertial sensors. The WiFi network is a campus network through TL-WR740N 150 M TP-LINK wireless router access. Software is written in C++ and objective-C using the OpenCV library. The Unity 3D engine is chosen to render 3D models.

### 4.1. Recognition Performance

**Figure 6:**
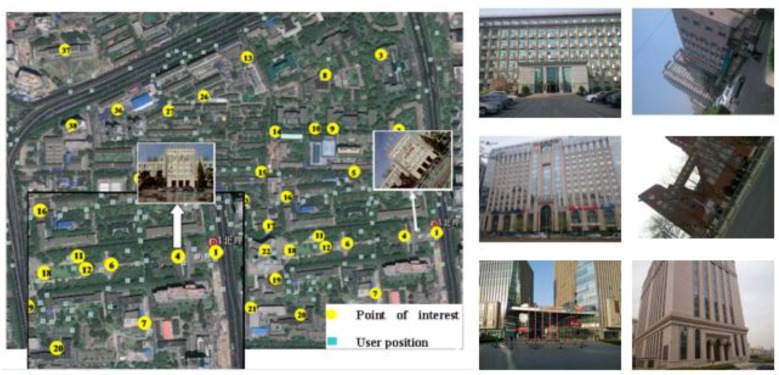
Examples of our database images.

To evaluate the performance of our scene recognition algorithm for a wide area, our own database (Figure 6 shows a part of our database) is chosen to test the recognition rate. Our database contains 4 K keyframe images selected from 800 outdoor scenes with partial occlusions, different viewpoints, scale and illumination changes. Each image is labeled with UTM GPS coordinates and gravity information (e.g., latitude: 39.96339504 longitude: 116.30417682; gx = 9.319382, gy = 0.66389465, gz = −2.3644562). The database is publicly available [45] and readers can download it by citing this paper or contacting the corresponding author by E-mail.

**Figure 7:**
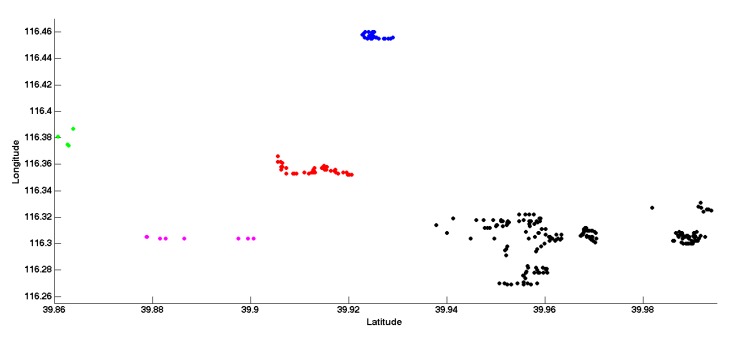
Geometry clustering result.

We apply the density-based clustering method to our database and Figure 7 shows the clustering results. In this experiment, we set radius as 0.03 and minimal points as nine so as to get 10 clusters. In order to demonstrate the results clearly, we only take five clusters as an example.

**Figure 8:**
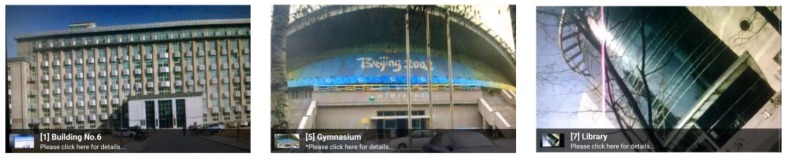
Recognition results.

Table 1 shows the recognition accuracy of our Geo-based GVLAD method compared with the state of the art approaches. The performance of different methods is measured by recall @ R which is defined as the proportion of query vectors for which the correct match is ranked within the top returned results. We use SURF features here for recognition and use PCA to reduce the dimensionality of the training dataset. In Table 1, K means the number of code words. As can be seen from Table 1, the retrieval accuracy can increase significantly with the help of GPS and gravity information. The retrieval accuracy of geo-based VLAD is about 4% higher than that of original VLAD. However, the performance of GVLAD is about 9% higher than original VLAD and a 6% improvement over that of geo-based VLAD. The retrieval accuracy of geo-based GVLAD shows an improvement of up to about 14%. From the experimental results we can see that using gravity information or GPS alone can improve the accuracy of VLAD and gravity helps more. The retrieval accuracy can increase a lot when using the method presented in this paper, with the help of both GPS and gravity information. In addition, the size of the recognition algorithm is about 5.99 MB when K is 128 and the average size of 3D structure is 0.68 MB for each scenario, which we can easily pre-download to the mobile phone. It is worth noting that partial occlusion of the buildings by the pedestrian and cars causes the distribution of features to change and thus affects the recognition results. Moreover, buildings that have similar color and symmetrical structure of the windows and doors also cause the percentage of correct matches to decrease. Figure 8 shows the recognition results of our method.

**Table 1:** The recognition accuracy.

Method	K = 64	K = 128	K = 256
VLAD	0.778	0.794	0.806
Geo-based VLAD	0.814	0.833	0.847
GVLAD	0.875	0.893	0.897
Geo-based GVLAD	0.922	0.933	0.934

### 4.2. Hybrid Tracking Performance

**Figure 9:**
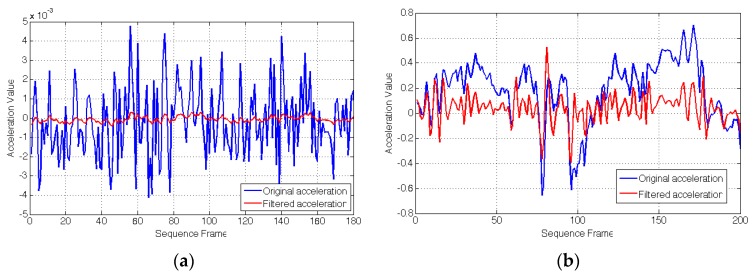
Effect of de-noising. (**a**) Static; (**b**) Random moving.

In our experiment, we firstly use wavelet de-noising with four layers to reduce the noise of the accelerometer data. Figure 9 shows the effect of de-noising on acceleration compared with the original acceleration. In Figure 9, the blue line shows the original acceleration and the red line shows the acceleration after denoising. Figure 9a gives the acceleration when the mobile device is stationary, and Figure 9b gives the acceleration when the mobile device moves randomly. Here, the change of X-axis is taken as an example. From Figure 9, we can see that the effect of wavelet threshold denoising is obvious for the accelerometer, but the sensitivity of the accelerometer to motion is slightly reduced.

**Figure 10:**
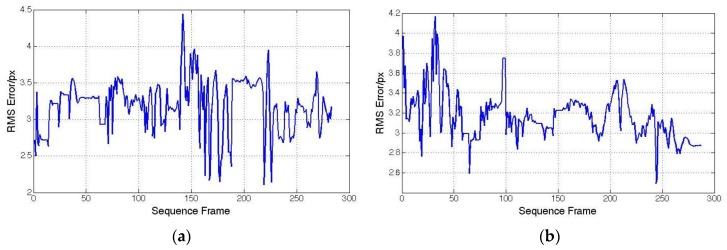
Re-projection error of hybrid tricking method. (**a**) Rotate along Y-axis; (**b**) Move backwards and forwards.

Because of the lack of ground truth data, we use the re-projection errors to test the accuracy of our hybrid tracking method. The re-projection error we utilized here is the squared distance between the projection of feature points in the current image and the measured 2D coordinates in the keyframe. Figure 10a gives the re-projection errors of the hybrid tracking method when a user holds an iPhone in his hand walking around randomly, with the iPhone is rotating along the Y-axis. The purpose of this kind of movement is to simulate the case when users make large view angle changes. Figure 10b gives the re-projection errors of the hybrid tracking method when users move backwards and forwards to simulate the case when users move close to or far from the scene. All the errors above are below 4.5 pixels, which demonstrates the accuracy of the proposed method.

**Figure 11:**
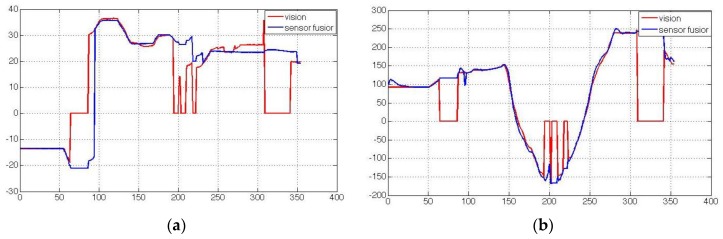
Motion estimation results when vision measurements are unavailable in some frames. (**a**) Rotation; (**b**) Translation.

Figure 11 shows the motion estimation results in comparison, when vision measurements are unavailable in some frames. Here, the change of X-axis is taken as an example. The red line shows the vision measurements. Some equaling zero correspond to missing vision data. The blue line shows motion estimation results by using the sensor fusion model method, where the pose is solely based on inertial sensors when vision data is unavailable. We can see in this figure that for a short time the camera pose can still be estimated accurately without vision data by using inertial data. However, without vision data the inertial data will quickly drift within several minutes.

**Figure 12:**
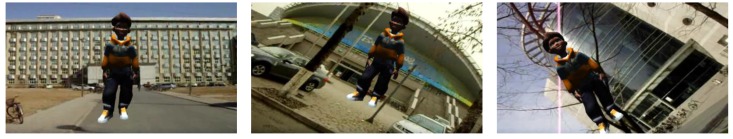
Tracking effects.

Figure 12 shows the tracking effects. We can see that the computer-generated 3D model is superimposed on the live image successfully.

### 4.3. Computation Time

In our experiments, for each keyframe and input image, we extract about 400 ORB features. The computation time of the experiment are recorded in Table 2.

**Table 2:** The average computation time.

Step		Time (ms)
Initialization phase	Feature Extraction	88.4
	Feature Matching	3.4
Tracking phase	Optical Flow Tracking	17.1
	PROSAC	2.5
	Pose Estimation	6.4
	Sensor Fusion (Prediction)	0.5
	Sensor Fusion (Correction)	1.4
	Render latency	0.5

As can be seen from Table 2, the time for feature extraction is 88.4 ms and for feature matching only 3.4 ms, which belong to the initialization phase. Next, Optical Flow Tracking and the PROSAC method take 19.6 ms. Finally, pose estimation needs about 6.4 ms and sensor fusion needs only 1.9 ms. It is worth noting that the render latency for the mobile device is only 0.5 ms in our experiment. Table 2 shows that our algorithm can meet the real-time requirements of mobile devices.

## 5. Conclusions

This paper describes a sensor-aware large-scale scene recognition and tracking algorithm applied for mobile augmented reality systems. A geo-based GVLAD method, which uses GPS and gravity information to improve the performance of recognition, is utilized to recognize different scenarios. An affine invariant interest point detector is used to extract natural features in the unprepared environment and track them frame-to-frame by computing the optical flow. Gyroscope and acceleration data from inertial sensors and vision are fused together to achieve significant improvements in tracking stability and robustness.

Experimental results demonstrate that our method is real-time, robust and effective in outdoor environments. However, our algorithm still has some limitations which require further improvement in future work. First, the stability of our system will drop quickly when the actual illumination conditions are quite different from the light conditions used in the training stage. The reason is whether an object that can be reliably detected mainly depends on the training images. Once trained, the performance can no longer be improved. Second, when the camera moves to some distant locations, the appearance of the selected features may be drastically different. This will lead to a sharp drop in the number of the inners. Third, due to the limited accuracy of mobile accelerometer, tracking algorithm will drift quickly in several minutes without vision data.
